# A Critical Review on Polymeric Biomaterials for Biomedical Applications

**DOI:** 10.3390/polym13173015

**Published:** 2021-09-06

**Authors:** Cheirmadurai Kalirajan, Amey Dukle, Arputharaj Joseph Nathanael, Tae-Hwan Oh, Geetha Manivasagam

**Affiliations:** 1 Centre for Biomaterials, Cellular and Molecular Theranostics (CBCMT), Vellore Institute of Technology (VIT), Vellore 632014, Tamil Nadu, India; cheirmadurai.k@vit.ac.in (C.K.); amey.dukle@vit.ac.in (A.D.); geethamanivasagam@vit.ac.in (G.M.); 2 School of Chemical Engineering, Yeungnam University, Gyeongsan 38541, Korea

**Keywords:** smart biomaterials, tissue engineering, polymers, hydrogels, shape memory, 3D printing

## Abstract

Natural and synthetic polymers have been explored for many years in the field of tissue engineering and regeneration. Researchers have developed many new strategies to design successful advanced polymeric biomaterials. In this review, we summarized the recent notable advancements in the preparation of smart polymeric biomaterials with self-healing and shape memory properties. We also discussed novel approaches used to develop different forms of polymeric biomaterials such as films, hydrogels and 3D printable biomaterials. In each part, the applications of the biomaterials in soft and hard tissue engineering with their in vitro and in vivo effects are underlined. The future direction of the polymeric biomaterials that could pave a path towards successful clinical implications is also underlined in this review.

## 1. Introduction

Tissue engineering focusses on the repair and restoration of the structural function in the damaged tissue. Tissue repair can be aided with the help of different materials, such as decellularized matrix, cells, scaffolds, and others [1,2]. In tissue engineering, scaffolds are most successfully used to achieve better regeneration. A scaffold is a three-dimensional platform which can mimic the real tissue environment and facilitates the attachment and proliferation of the cells [3]. The material selection for the designing of a scaffold for tissue engineering has some criteria concerning, e.g., their biocompatibility, wettability, biodegradability, etc. Biomaterials are engineered materials with the ability to interact with biological systems to aid the processes of repair and regeneration. The use of polymers in the preparation of biomaterials has been practiced for more than half a decade. The wide range of chemical and functional properties of the polymers allows researchers to create an oriented structure for specific biomedical applications [4,5]. The polymers used for the preparation of the biomaterials fall under two categories, namely natural or synthetic polymers.

**Figure 1:**
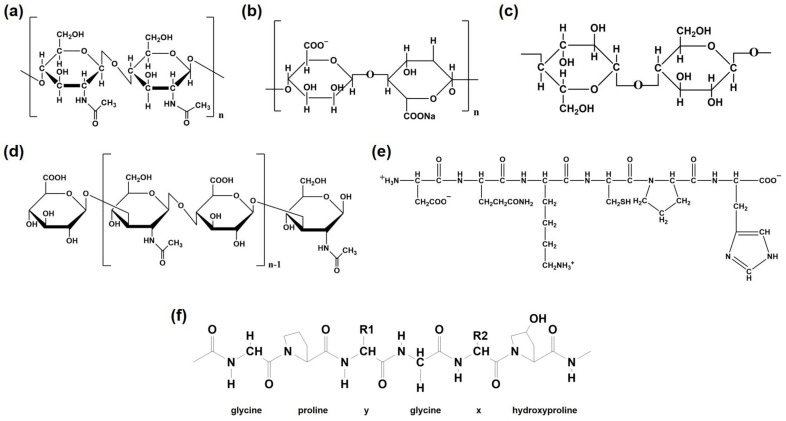
Chemical structure of natural polymers (**a**) Chitin, (**b**) Sodium alginate, (**c**) Cellulose, (**d**) Hyaluronic acid, (**e**) Gelatin, and (**f**) Collagen.

Natural polymers have been used in the preparation of biomaterials for a long time due to their biocompatibility and low immunogenic properties. Collagen is a natural protein present in the skin and other connective tissues. Collagen has been widely used in the preparation of scaffolds and implants for tissue engineering and regeneration [6,7,8]. Other than collagen, natural polymers like fibrin, keratin, fibronectin, and laminin are also explored for their potency in the field of tissue engineering [9,10,11,12]. Natural polysaccharides like alginate, chitin, chitosan, and different gums are also studied most for their ability to use as a biomaterial [13,14]. Natural polymers have some limitation in their application as biomaterials due to their rapid degradation property. Natural polymers also present many challenges, including their low mechanical property and high risk of contamination with microbial species like bacteria and fungi. Some people can also develop an allergic reaction to natural polymers since they are from different animal sources. The large-scale synthesis of natural polymers is also another tough target and their property customization is also difficult [15]. Figure 1 shows the chemical structures of some natural polymers used in biomedical applications.

**Figure 2:**
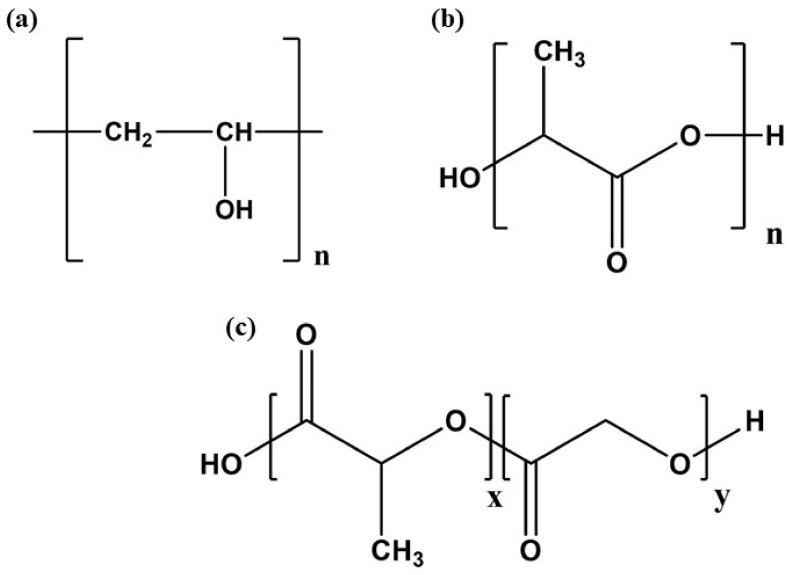
Chemical structure of synthetic polymers (**a**) Polyvinyl alcohol (PVA), (**b**) Polylactic acid (PLA), and (**c**) Poly(lactic-co-glycolic) acid (PLGA).

**Scheme 1:**
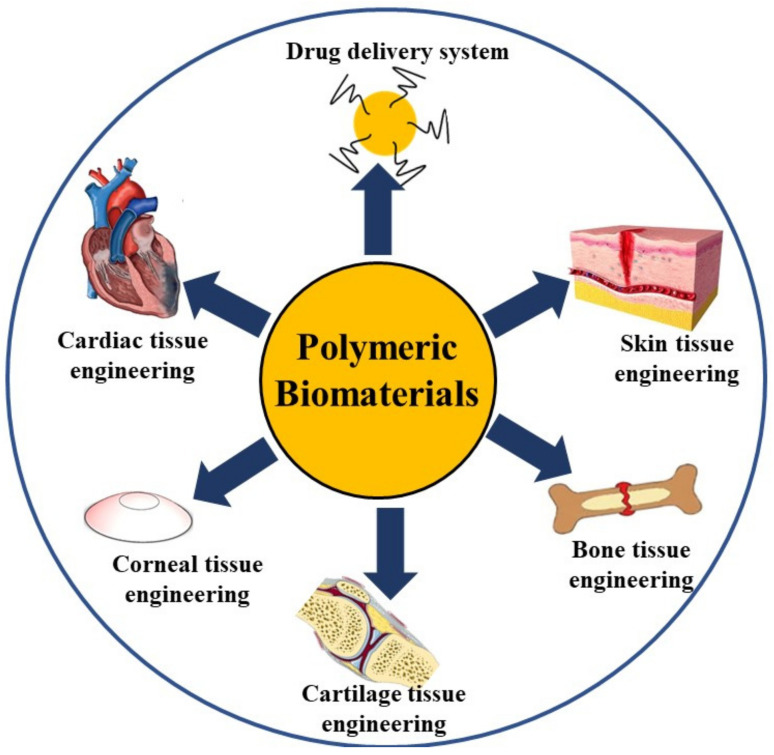
Schematic illustrating the applications of polymeric biomaterials in different biomedical field.

Synthetic polymers are the best substitutes for patients with allergic reaction to natural polymers. Synthetic polymers are less immunogenic and do not cause chronic immunogenic inflammation. Synthetic polymers are also known to have better mechanical properties when compared to the natural polymers [16]. The biodegradability of the synthetic polymers can also be adjusted and this makes them suitable for tissue engineering applications. Several synthetic polymers, e.g., polylactic acid (PLA), polyvinyl alcohol (PVA), polycaprolactone (PCL), and polylactic-co-glycolic acid (PLGA), are the most studied due to their potency in biomedical applications [17,18,19,20]. Natural and synthetic polymers can be prepared in different forms, e.g., films, 3D scaffolds, hydrogels, and 3D printed implants [21,22,23,24]. The polymeric biomaterials can be loaded with different active molecules like polyphenols to improve their potency in tissue regeneration [25]. The chemical structures of some biodegradable synthetic polymers most commonly used for the preparation of biomaterials are shown in Figure 2. We have inserted a scheme (Scheme 1) to illustrate the different applications of the polymeric biomaterials in the field of biomedical and tissue engineering. 

## 2. Different Forms of Natural and Synthetic Smart Polymeric Biomaterials

### 2.1. Polymeric Films

Polymer based films were introduced in the tissue engineering field initially due to their easy preparation method. Slowly, the polymeric films have been shown to demonstrate potency for use in occlusive wound dressings in the field of wound management. Natural and synthetic polymer-based films have been studied extensively as a wound dressing material [26,27,28]. The polymeric film dressings fall under two categories, namely passive or interactive. Natural polymers-based films are interactive and occlusive. However, the synthetic polymer films may be passive or interactive. The passive or non-occlusive dressings are used to cover the wound alone. Interactive or occlusive polymer films act as a barrier for microorganisms and they also facilitate wound healing [29]. Polymer based films can absorb wound exudate and maintain a moist environment around the wound bed. The moisture environment helps in healing and regeneration. The polymeric film will also allow an air passage in and out of the wound bed [29]. Natural polymers, like collagen, chitosan, carboxymethyl cellulose, and alginate, are the most studied for film preparation and their application as wound dressing materials [26,27,30,31]. Synthetic polymers, like PVA, PEG, and PCL, have been reported for their potential application as wound dressing materials [30,32,33]. Natural and synthetic polymers are used as individual components or prepared as a composite to improve their mechanical properties.

**Figure 3:**
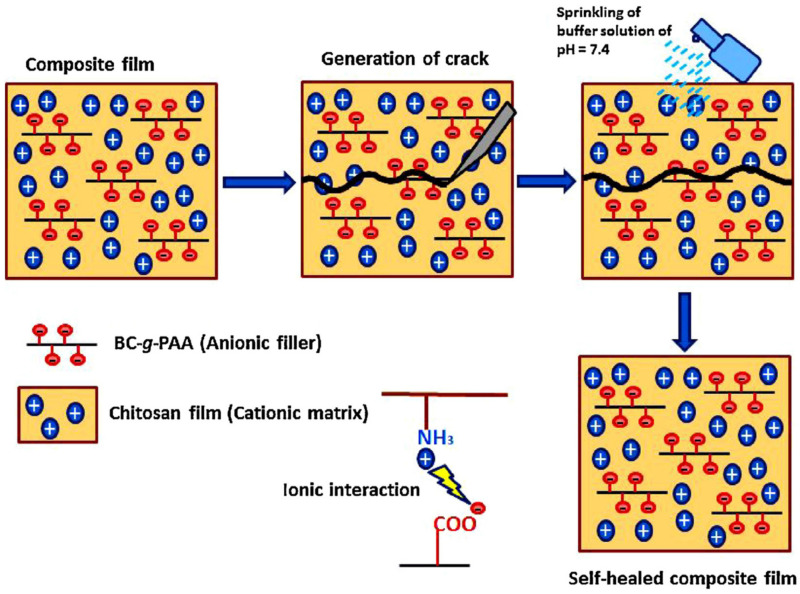
The simple schematic shows the self-healing mechanism of cationic chitosan matrix assisted by anionic filler (Poly(acrylic acid) grafted bacterial cellulose). (Reproduced with permission from [43] Copyright 2017, Elsevier).

The films based on natural polymers are known to have less structural stability and easily degradable properties. To improve their physical properties, chemical cross-linkers like glutaraldehyde were used initially. In recent years, the chemical cross-linkers have proven to be cytotoxic for the proliferating cells and researchers have started to explore non-cytotoxic cross-linkers [34]. Different cross-linking methods, like physical cross-linking and ionic cross-liking, were used to prepare a polymeric film for biomedical applications [35,36]. The polymeric films loaded with drug, natural active molecules like curcumin and quercetin, and metal nanoparticles like zinc and silver have been reported with improved antibacterial and healing properties [37,38,39,40,41,42]. The polymeric films loaded with active molecules have been used for the controlled release through the transdermal route. In recent years, the development of materials with dynamic self-healing ability is getting more interest due to their restoring capacity even after severe deformation. Poly(acrylic acid) grafted bacterial cellulose (BC-g-PAA) based self-healable polyelectrolyte film was reported by a research group for the wound dressing applications [43]. The self-healable property of the prepared films was studied under two different pH conditions (pH = 7.4 and pH = 5.5). Single or multiple notches were generated on the film and the buffer solution was sprinkled over the film. The developed film has shown self-healing property at both pH = 7.4 and pH = 5.5. Self-healing can occur through the permanent covalent bond formation or ionic bond formation. In the composite films, when the buffer solution was sprinkled with the cationic chitosan, molecules were observed attempting to diffuse through the buffer solution and form an ionic interaction with the anionic filler, i.e., modified bacterial cellulose. The schematic clearly shows the self-healing mechanism of the prepared composite film (Figure 3).

**Figure 4:**
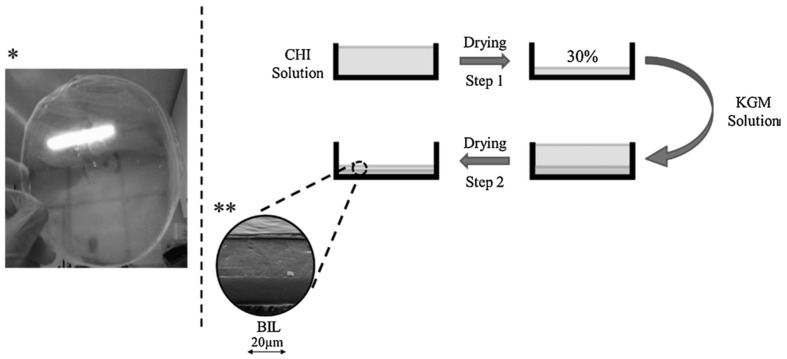
Picturized representation of the Two-step casting process used for the production of bilayer films; * Digital image of the prepared bilayer film; ** shows the SEM image of the bilayer film. (Reproduced with permission from [44] Copyright Elsevier).

Layer by layer assembly of the films has been become very popular due to the low-cost preparation methods and potency for use in the controlled release of growth factors, among other advantages. A research group has developed a bilayer (BIL) structure using chitosan (CHI) and konjac glucomannan (KGM) to preserve the intrinsic property of each polymer on the different layers [44]. Figure 4 shows the preparation of a bilayer structure. The authors have investigated the water vapor transmission rate, swelling, solubility, and mechanical property of the bilayer structure to understand the interface interaction between the polymers. Alginate-based bilayer film has been developed by a group of researchers for the controlled slow release of a loaded drug for wound healing applications [45]. The upper layer is loaded with a model drug and the bottom layer drug-free, acting as a release rate controlling membrane. These authors have observed that the bilayer structure shows controlled release over the single layer films and also shows a high rate of healing with well-defined epidermis compared to the control results.

The preparation of electrically conductive polymeric biomaterials is also getting more interest in the field of tissue engineering and regenerative medicine. A conductive polymeric film based on sodium alginate and gelatin was developed for wound dressing applications [46]. Reduced graphene oxide was mixed with polymers introduce the electrical conductivity property to the polymeric film. The results showed that the polymeric films with the conductive property had good cell attachment and viability.

### 2.2. Polymeric Sponges

Recent studies show the importance of the porous nature of the scaffolds used in the tissue engineering applications. The porous structure facilitates the cell attachment, proliferation, vascularization and ECM deposition. The porous scaffolds also help in exudate absorption and maintain a moist environment in the wound bed. It also facilitates gas exchange and nutrient transport through the interconnected channel network [47]. The ordered porous structure also enables easy drug loading and controlled release [48]. The 3D porous scaffolds provide a 3D structure for the cell attachment, proliferation and differentiation. This has prompted researchers to study the cell–matrix interactions in a 3D cell culture model [49,50]. Freeze drying is the common method used for the preparation of porous scaffolds for biomedical applications. A more porous structure may also affect the final mechanical property of the scaffolds. The ideal pore size required for skin and bone tissue engineering varies. From the earlier studies, it was found that the pore size ranging from 200 to 400 µm was suitable for bone tissue engineering [51], whereas the pores ranging from 50 to 200 µm were found to be effective in the smooth muscles and soft tissue engineering process [52,53,54]. Natural and synthetic polymers-based sponges have been studied for their potential biomedical applications. The sponges prepared from the natural polymers are known to have low mechanical strength and preparing them as a composite with the synthetic polymer is getting interest. Researchers are also exploring different cross-linking methods to improve the mechanical strength and pore size distribution of the scaffolds.

**Figure 5:**
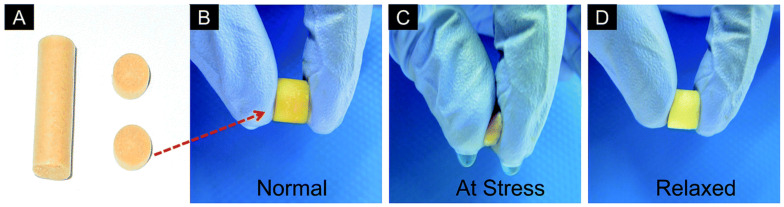
3D biocomposite agarose–chitosan scaffold, (**A**) in various shapes, which displays sponge-like characteristic in (**B**) normal (**C**) at stress and (**D**) relaxed conditions. (Reproduced with permission from [55] under creative commons CC-BY license, 2015).

A 3D biocomposite macroporous scaffold based on agarose and chitosan was prepared for an in vitro 3D liver tissue model [55]. The prepared 3D scaffold was evaluated for their applications in pre-clinical therapeutic testing. The prepared scaffolds show interconnected pore structures with an average size ranging from 40 to 70 µm. From the rheological studies, it was observed that the hydrated scaffolds exhibited sponge like visco-elastic behavior without any deformation in the shape. These authors have also tested the elastic property of the scaffold using a simple press and release method, as shown in the Figure 5. The in vitro results show that the neutral pH enhances the cell–cell interfacial interaction and hepatocyte colonization.

Many research efforts have been made to prepare a scaffold with uniform pore size distribution. Researchers have developed a method based on ice particulates and freeze-drying to prepare a gelatin scaffold with homogenous pore structure and improved mechanical property [56]. The scanning electron microscopic images of the scaffolds prepared using 70% ice particulates showed one open pore structure with the macropores interconnected by micropores on their wall, whereas the control group showed the lamellar structure with less interconnected pore structure. From their results, it is clear that gelatin scaffolds prepared using 70% ice particulates aided the homogenous seeding of bovine articular chondrocytes and the formation of cartilage extracellular matrix. The gelatin scaffolds prepared without ice particulates did not show better results than the scaffolds prepared with ice particulates.

Natural and synthetic polymers-based 3D scaffolds/sponges have wide applications in skin and bone tissue engineering [57,58]. The scaffolds can be loaded with active molecules or drug to improve their antibacterial and wound healing properties [59]. Metal nanoparticles are also attracting significant interest as an active material for biomedical applications [60,61]. Natural and synthetic polymers can be taken together to prepare them as a composite scaffold. A biodegradable composite 3D scaffold based on fibroin/chitin/silver nanoparticles was prepared and its antimicrobial property was evaluated by a group of researchers [62]. The prepared nanocomposite scaffolds had good biocompatibility, antimicrobial and mechanical property. The nanocomposite scaffolds exhibited antimicrobial properties against common bacterial wound pathogens, e.g., *Escherichia coli*, *Staphylococcus aureus,* and a fugal species *Candida albicans*. In another study a bioactive wound dressing based on collagen loaded with zinc oxide nanoparticles was developed [63]. The potency of the prepared bioactive scaffolds on the healing of the chronic burn wounds was evaluated. From the animal trials, it was shown that the prepared scaffolds were able to heal the wound on the 21st day of treatment. The authors claimed that the antibacterial activity imparted by ZnO nanoparticles and high regenerative potency were due to the presence collagen, supporting faster healing of the chronic burns. Natural polymers are known to have low mechanical property. The composite scaffolds made with natural and synthetic polymers are also investigated in tissue engineering due to their improved physical and mechanical properties. In another report, gelatin/PVA based 3D porous scaffolds were prepared with improved mechanical property and their cytocompatibility was evaluated [64].

**Figure 6:**
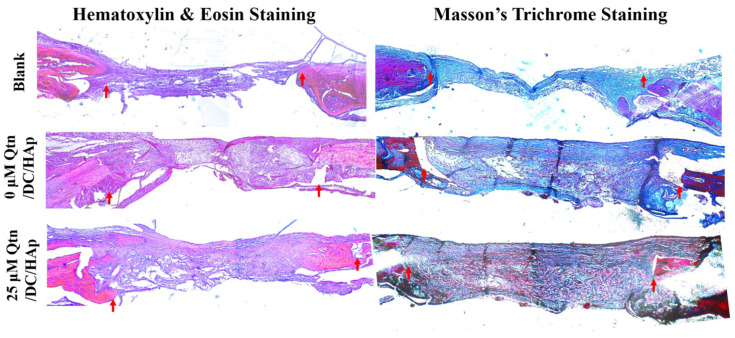
Histological evaluation of calvaria collected after 8 weeks of treatment with quercetin/collagen/hydroxyapatite sponges. (Red arrow indicates the edge of the defect at magnification ×40) (Reproduced with permission from [65] Copyright 2020, Wiley).

The 3D porous scaffolds are also explored in the field of bone and cartilage tissue engineering for their applications. In a recent study, authors have attempted to develop a collagen/hydroxyapatite sponges loaded with quercetin for enhanced bone regeneration [65]. The effect of quercetin on osteogenesis was evaluated by culturing the bone marrow stem cells on the sponges. They have also carried out in-vivo studies to confirm the bone regeneration property of the scaffolds. Histological staining was carried out to evaluate the bone regeneration capacity of the prepared scaffolds on the animal calvaria defect model. The hematoxylin & eosin (H&E) staining images clearly show the presence of calcified bone in the defective area, whereas the blank group did not show any visible bone formation (Figure 6). In the Masson’s trichrome staining, new bone formation appeared in red, and it was high in the sponge with 25 µm quercetin concentration (Figure 6).

In another report, the research group has studied the effect of chitosan sponges with different degrees of deacetylation on the attachment and differentiation of primary human osteoblasts [66]. From the in vitro study, they observed that chitosan sponges with higher deacetylation showed good cell spread and differentiation. They carried out an alkaline phosphatase (ALP) assay and observed that the chitosan sponges with higher deacetylation showed increased ALP activity when compared to sponges with lower deacetylation. They also quantified different bone markers and cytokines in the culture medium. From the quantitative results, they noticed that the chitosan sponges with lower deacetylation induced the expression of sclerostin and osteoprotegerin. They have reported that chitosan sponges with different deacetylation and molecular weight respond differently to the guided bone regeneration. Synthetic polymers like PVA and PCL in combination with natural polymers have been extensively studied for their potency in bone tissue engineering applications. The polymeric sponges also used as a carrier for the loading and controlled release of the drug. The polymeric sponges with high pore volume and porosity makes the drug loading and release possible. In order to achieve controlled release of the loaded drug, an external polymer coating is used in some studies [48].

### 2.3. Hydrogels

Hydrogels can be defined as a 3D network of natural or synthetic polymers, which can absorb and hold a greater amount of water [67]. The polymeric network is formed by cross-linking polymeric chains with covalent or non-covalent interactions. The cross-linking of the hydrogels can also be performed by adding the ions like Ca^2+^, Zn^2+^, and Mg^2+^ to the polymeric monomers. The ions can induce the gelation through forming ionic interactions with the polymeric chains [68,69]. The hydrogels can be categorized into various types based on the polymers used (natural or synthetic) and the method used (physical or chemical cross-linking) for the hydrogel preparation. The swollen 3D structure of the hydrogels makes the cells to attach and diffuse easily. The hydrogels have a similar structure to the natural soft tissues, which encourages their potential use in the field of tissue engineering and regenerative medicine. Hydrogels have been extensively studied for their applications in drug delivery, tissue engineering, 3D tissue cultures, and contact lenses [70,71,72,73]. Hydrogels were prepared by using the cross-linked hydrophilic monomers that can absorb water.

**Figure 7:**
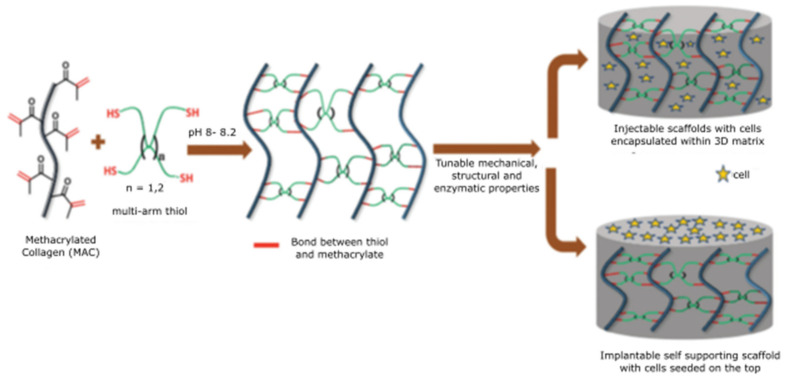
Schematic shows the fabrication of a methacrylated collagen (MAC) hydrogel cross-linked with PEG thiols (4- and 8-arms). (Reproduced with permission from [74] Copyright 2015, The Royal Society of Chemistry).

Collagen is the most extensively studied natural polymer for the hydrogel preparation and their biomedical applications. Researchers are making more effort to improve the mechanical properties among others, e.g., antibacterial, without disturbing the secondary structure of the collagen. In a study, authors attempted to develop a type-I collagen hydrogel without affecting its triple helical confirmation [74]. They functionalized collagen with methacrylation and then reacted with PEG-thiols to obtain a biocompatible hydrogel with tunable properties (Figure 7). To confirm the unaltered triple helical confirmation of the collagen after functionalization, they carried out CD spectroscopic analysis. From the CD spectroscopic results, they observed that the functionalized collagen shows a clear positive peak and a negative peak like the pristine collagen solution. They studied the effectiveness of the hydrogels in 3D cell encapsulations using two different systems. In a first model, they seeded human corneal epithelial cells on the top of hydrogels and their proliferation was evaluated. They observed that the prepared hydrogels were biocompatible and on day 1, and they demonstrated good attachment and proliferation of the cells. On day 5, the cells were well confluent, and the results were comparable to the tissue culture polystyrene and pristine collagen hydrogels cross-linked with EDC.

In the second model, they adopted faster gelling formulation incorporated with cardiac progenitor cells. They carried out live-dead cells assay to evaluate the cell viability inside the hydrogel matrix. They reported that the cells were highly viable after three days of seeding and they have also observed well spreading and elongated morphology of the cells. The hydrogels started to degrade after five days and released the encapsulated cells into the media. Their results show that the soft hydrogels can be effectively used as the delivery system of cardiac progenitor cells into the heart.

**Figure 8:**
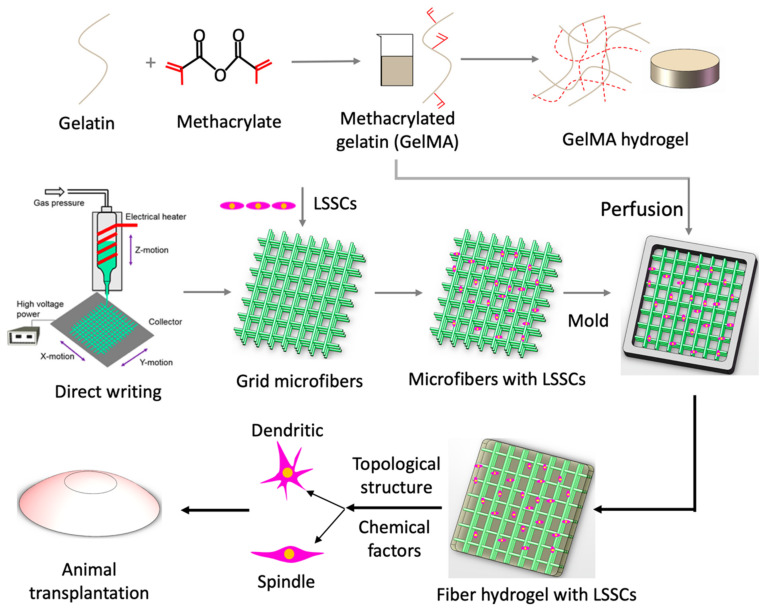
The schematic illustration showing the preparation of fiber reinforced GelMA hydrogel for of the regeneration of the damaged corneal stroma. (Reproduced with permission from [75] under creative commons CC-BY license, 2020).

Hydrogels have been prepared from natural polymers known for their application in corneal defects due to their high aqueous environment, biocompatibility, and high transparent nature. To overcome the low mechanical stability of hydrogels, chemically functionalized polymers have recently emerged. In an attempt, researchers have developed poly(ε-caprolactone)-poly(ethylene glycol) (PECL) sub-micro fibers reinforced gelatin methacyrylate (GelMA) hydrogel [75]. They have used the direct writing method to prepare PECL sub-micro fibers, and GelMA solution was infused to prepare a fiber hydrogel. The fiber reinforced hydrogels were inoculated with limbal stromal stem cells (LSSCs) and they were found to be differentiated into keratocytes and to maintain their phenotype. The schematic for the preparation of fiber reinforced GelMA hydrogel is shown in Figure 8. The authors studied the potency of the hydrogel in vivo using a rabbit model. The prepared hydrogels were proven to be useful in the regeneration of the damaged corneal stroma.

Natural polymers are well-known to have low mechanical property and easy degradability. Many research attempts have been made to improve their properties during preparing them as a hydrogel. Natural polymers can be mixed with other polysaccharides and synthetic polymers to prepare a composite hydrogel with improved properties. In a study collagen/polyacrylamide hydrogel with high toughness and good adhesive property was prepared and used as a wound dressing material [76]. The authors have used dopamine grafted sodium alginate as a cross-linker to improve the adhesive property of the hydrogel. The prepared collagen/polyacrylamide hydrogel was found to be biocompatible and it also helps in better wound healing. Hydrogels have also been investigated for their applications in the area of bone tissue engineering. Yang et al. developed a hyaluronic acid/collagen hydrogel conjugated with icariin for osteochondral repair [77]. The in vitro results demonstrate that the bone marrow stem cells encapsulated in the hydrogels show better osteogenic and chondrogenic differentiation.

Wound healing is a complex process and the current treatment strategies used for the drug delivery systems were designed to treat certain phases and not every stage of healing. In order to address this problem, a multilayered hydrogel system that is capable of delivering the bioactive subtances sequentially at each healing stage was developed. The multilayer hydrogel system contains an injectable sodium alginate/Bioglass composite hydrogel carrying sodium alginate microsphere with cells (SAcm) [78]. The SAcm was encapsulated with poly(lactic-co-glycolic acid) (PLGA) microspheres containg pirfenidone. The release of biactive glass (BG) ionic products from the multilayer hydrogel during the first three days of healing would help in regulating the inflammation and initiating the subsequent process of the healing. The release of BG ionic products stimulates the polarization of macrophages into M2 phenotype, and thereby reduces inflammation. Then, the SAcm microparticles release the cells during the second phase of healing, which would help in the formation of vascularized granulation tissue formation. Finally, the release of pirfenidone from the PLGA microspheres helps in preventing the fibrosis and scar formation during the remodelling phase. The composition of the bioactive components in the multilayered hydrogel system can be modified and used for different tissue engineering applications.

## 3. Injectable Hydrogels

Injectable hydrogels represent an in-situ gelling system that requires a controlled gelation kinetics. In this system, a sol or pregel solution is injected into a targeting site to allow the gelation to occur. Injectable hydrogels are favorable in many biomedical applications, especially in drug delivery and tissue engineering due to their good carrier property in three dimensions. The injectable hydrogels are also attracting more attention due to their low invasive property and adapting shape in real time [79]. The injectable hydrogels have some basic criteria, like mild gelation conditions and timely gelation, for successful biomedical applications. Due to the low mechanical strength, the injectable hydrogels were found to mimic the structure of the soft tissues more than a hard tissue.

**Figure 9:**
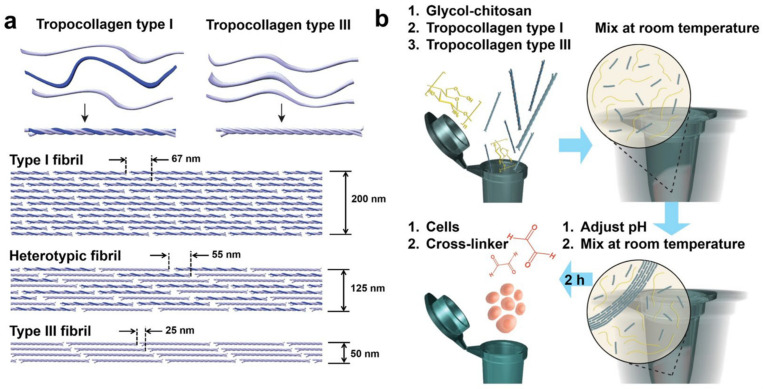
(**a**) Simple illustration of collagen type I and III, (**b**) Schematic representation of the step-by-step fabrication procedure of injectable hydrogel with tissue specific collagen ratio. (Reproduced with permission from [80] under creative commons CC-BY license, 2018).

Collagen type-I is the most common protein used in the preparation of biomaterials. Collagen type-III is also present in large quantities in many soft tissues along with type-I and not explored much for the preparation of biomaterials. Latifi et al. developed a novel injectable hydrogel with tissue specific collagen-I and III ratios [80]. The injectable hydrogel system is composed of collagen and glycol chitosan. They added the required amount of collagen type I and III and glycol chitosan to an Eppendorf vial and mixed at room temperature. The pH of the solution was maintained at physiological pH = 7.4 in the final cell seeded hydrogel (Figure 9). The prepared hydrogels showed a half lifetime for more than 35 days in the enzyme solution with great biochemical stability. The prepared hybrid hydrogels were mechanically stable under continuous cyclic loading and seem to represent a suitable candidate for the engineering of mechanically challenged soft tissues. Their hybrid hydrogels implanted with the fibroblasts that showed actin fibers after two days of culturing. The negative controls showed a rounded morphology after seven days of culturing. The prepared hydrogels might prevent or modulate the scar formation via enhancing Collagen I–III levels in the wounding site.

**Figure 10:**
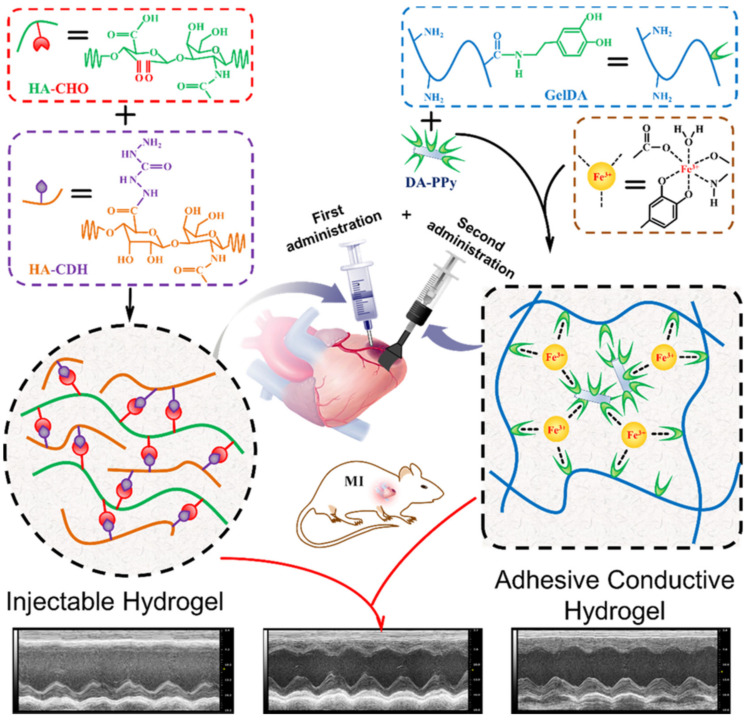
Schematic representation of the treatment of myocardial infarction using the coadministration of the adhesive conductive hydrogel patch and injectable hydrogel. (Reproduced with permission from [81]. Copyright 2019, American Chemical Society).

In tissue engineering strategies, injectable hydrogels and biomaterial cardiac patches have been used to treat myocardial infarctions. The therapeutic efficacy of the single system is very limited. Wu et al. have developed combined therapy of approach using the coadministration of the adhesive conductive hydrogel patch and injectable hydrogel for the treatment of myocardial infarction [81]. Here, they prepared hydrogels from the two natural polymers gelatin and hyaluronic acid. The injectable hydrogel used in this study was prepared via a Schiff base reaction between oxidized sodium hyaluronic acid and hydrazided hyaluronic acid. Cardiac adhesive patches were fabricated using Fe^3+^ induced ionic coordination between the mixture of dopamine-gelatin conjugates and dopamine-functionalized polypyrrole. The adhesive patches form a viscous fluid at the initiation of gelation, which can be directly applied on the surface of the myocardium to avoid surgical sutures. The schematic in Figure 10 clearly describes the combined injectable and adhesive hydrogel for the treatment of myocardial infarction. They have carried out animal studies using rats to evaluate the efficiency of the combination therapy in the treatment of myocardial infarctions. Their results demonstrated that the developed combined hydrogel strategy has prominent effects in improving cardiac function after the occurrence of myocardial infarctions.

**Figure 11:**
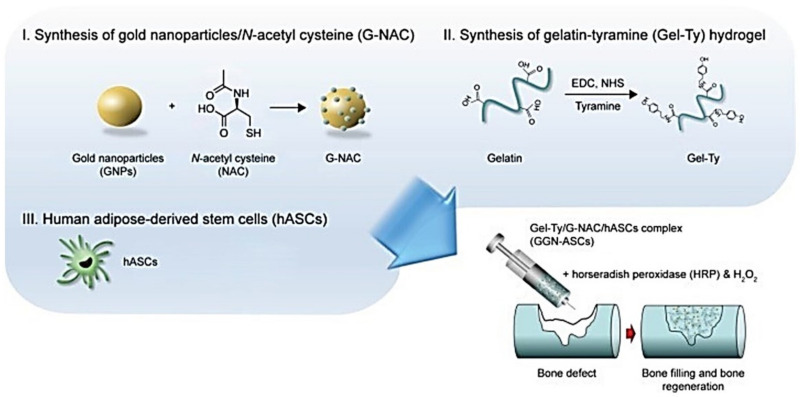
Schematic diagram shows the preparation enzyme cross-linked gelatin hydrogel for the bone regeneration applications. (Reproduced with permission from [82] under common creative CC-BY license, 2018).

The application of the injectable hydrogels in bone tissue engineering is also explored. In an attempt, researchers have developed an injectable hydrogel based on enzyme cross-linked gelatin [82]. The hydrogel was loaded with N-acetyl cysteine grafted gold nanoparticles (G-NAC). The schematic given in Figure 11 clearly explains the hydrogel preprations and their applications. They have characharaterized the gold nanoparticles, G-NAC, gelatin hydrogel for their biocompatibility and osteodifferentiation using human adipose derived stem cells. From their in-vitro results, they have proposed that the system can be used successfully for osteodifferentiation in bone tissue engineering.

## 4. Stimuli-Resposive Polymeric Hydrogels

Hydrogels are widely used in the biomedical and tissue engineering applications due to their efficient loading and release of active molecules and drugs. In recent years, stimuli responsive hydrogels have received increasing attention due to their controllable release of drug using external stimuli, such as temperature, pH, light, and ultrasound [83]. These stimuli responsive hydrogels are termed as smart polymeric drug delivery systems that are capable of controlled drug release and can protect the loaded drug from easy degradation. The polymers with lower critical solution temperature (LCST) will be the best choice for the preparation of thermal responsive hydrogels, since they form hydrogels only above its LCST. The sol-gel transition of these thermally responsive polymers at LCST closely resembles the folding of a protein under particular temperature [83,84,85].

Thermally responsive hydrogel based on chitosan and poloxamer 123 (CP) copolymers was developed for wound healing applications [86]. Gelatin was added to the hydrogels at different weight percentages to give a suitable stiffness required for the tissue engineering. Curcumin was loaded to the hydrogels and the efficiency of the developed hydrogels on the wound healing was evaluated. In this study, they prepared hydrogels with and without gelatin. The hydrogels with gelatin showed good swelling and curcumin release properties when compared to the hydrogels without gelatin. The in vivo results demonstrated that the CP-curcumin-gelatin hydrogels better results when compared to other groups. Such thermoresponsive hydrogels could represent an attractive polymeric biomaterial for tissue engineering and drug release applications.

In recent years, cell capturing and releasing in 3D biomimetic structures has received increase attention in the field of targeted therapy and tissue engineering. Microcarriers have emerged as an effective and novel 3D platform for the loading of the cells. Microcarriers have been developed using many methods, and microfluidics is the most recently developed, successful method. Wang et al. developed near-infrared (NIR) responsive graphene oxide (GO) hydrogel based microcarriers for controllable cell capture and release [87]. In this study, the authors developed GO microcarriers incorporated with poly(N-isopropylacrylamide) (pNIPAM) and gelatin methacrylate (GelMA) hydrogels. The developed hydrogel composite showed photothermal response due to the NIR response of the GO and thermally responsive shape transition of the pNIPAM. The biocompatibility of GelMA allows the cells to proliferate inside the microcarriers. The effect of developed hydrogel microcarriers was evaluated in mice tumor models. Their results demonstrated that the NIR responsive GO microcarriers facilitate tumor formation and angiogenesis in immunocompetent mice models. These results prove that the developed NIR responsive GO hydrogel carriers can be utilized in many biological and tissue engineering applications.

The pH responsive hydrogels are promising biomaterials in the field of tissue engineering and regeneration. Many research attempts have been made to prepare pH responsive hydrogels for various biomedical applications. Park et al. developed a pH responsive composite hydrogel using a natural and synthetic polymer namely carboxymethylcellulose and 2-hydroxyethyl acrylate, respectively [88]. The hydrogel was loaded with naringenin and efficacy for transdermal delivery was evaluated using in vitro characterizations. The drug release property of the hydrogel was evaluated at three different pH, i.e., pH = 5.5 (normal skin), pH = 7.5 (acne skin), and pH = 8.5 (atopic skin). The hydrogels showed maximum release at pH = 8.5 and this result suggest that the prepared hydrogels can be effectively used for treating the atopic dermatitis. Polydopamine (PDA) is a class of synthetic melanin with various interesting properties, including rich antioxidant activity. In recent years, many efforts have been made to develop stimuli-responsive, PDA-based smart materials [89,90].

## 5. Shape Memory Polymeric Materials

**Figure 12:**
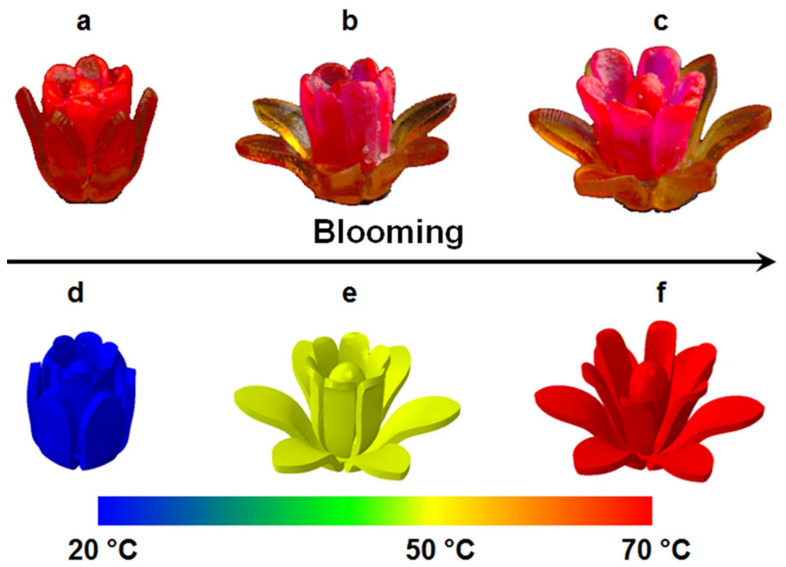
Schematic shows the different shapes of multimaterial flower at various temperature. The flower displays (**a**) bud stage at 20 °C, (**b**) opening of outer petals at 50 °C and (**c**) completely bloomed at 70 °C. (**d**–**f**) FE simulations of the corresponding flower blooming process. (Reproduced with permission from [95] CC-BY License 2018).

Shape memory polymers (SMP) denote a class of polymers capable of ‘memorizing’ a permanent or temporary shape [91]. SMPs undergo temporary shape change upon exposure to an external stimulus. When the stimulus is removed, they regain their original shape (Figure 12). The external stimuli range of temperature, light, pH, moisture to electric and magnetic field [92,93]. SMPs have been widely probed for applications, such as self-sealing components in intelligent packaging, self-healing, drug delivery, biosensors, biomedical devices, microsystem components, and smart textiles [94]. Biodegradable SMP are also explored as temporary use implants to avoid additional incision procedures [94]. Due to their higher strain recovery, improved compliance matching, biodegradability, and drug delivery capabilities, SMP are also being explored for stent applications. Further, a 4D printing approach of SMP is receiving more interest to create a high-resolution shape memory architecture. By controlling the dynamic properties of different SMPs used in the structure, a research group has designed a material with time-dependent shape recovery. They have demonstrated the sequential shape recovery of a multimaterial flower at different temperatures [95]. Further, we discuss a few notable works in biomedical applications of SMPs.

Shape memory polylactide (PLA) have been explored for the development of a 3D printed vascular stent from a biodegradable SMP [96]. The stent is initially compressed to a smaller diameter and inserted into the body. Once inside the body, the stent is heat triggered and expanded to its final shape. The stent showed excellent shape stability at room temperature and also allowed for personalised design using 3D printing. With better control over the trigger temperature, these stents can potentially replace the existing metal based vascular stents.

SMPs have also been probed for use in intracranial aneurysm coils. The coils are proposed to replace the platinum coils currently used for the treatment. Due to bio-inertness, the platinum coils experienced a significant failure rate in the treatment. The coils fabricated using Calomer, a propriety SMP produced by The Polymer Technology Group, Inc, was investigated in the study [97]. In the test environment, the coils expanded to the programmed shape when in contact with hot flowing water. The hydrodynamic forces also did not have any effect on the shape recovery. With the coils being stable inside the aneurysm dome and the coil not migrating within the cavity, the study showed promising results for further studies to be conducted.

Solution stimulated injectable antibacterial conductive cryogels with shape memory property are being explored for hemorrhage homeostasis and wound healing [98]. The cryogels are prepared using carbon nanotube (CNT) and quaternized chitosan functionalized with glycidyl methacrylate. Chitosan films cross-linked with epoxy polymers are also explored for production of biodegradable vascular stents [99]. Compared to metallic stents, the stent shows higher elasticity as well as rapid expansion when subjected to hydration. Preliminary animal studies have pitted these stents as a superior alternative to metallic stents owing to their biodegradability and drug delivery capabilities.

**Figure 13:**
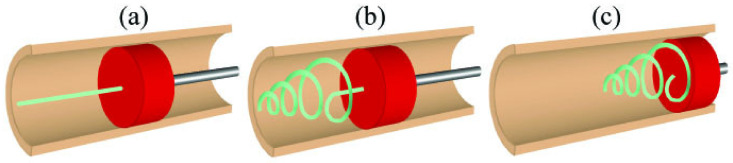
Representation of endovascular thrombectomy using the laser activated shape memory polymer micro actuator attached to an optical fiber. (**a**) In its secondary straight rod form, the micro actuator is delivered through a catheter distal to the thrombotic vascular occlusion. (**b**) Primary corkscrew form of micro actuator induced by laser heating. (**c**) The deployed micro actuator is retracted to capture the thrombus. (Reproduced with permission from [100] Copyright 2005, The Optical Society).

Light stimulated SMPs fabricated from thermoplastic polymers (MM5520 and MM6520) with a glass transition temperature of approximately 65 °C are explored for the fabrication of an intravascular thrombectomy device (Figure 13) [100]. Laser light with 810 nm wavelength was delivered by an SMP micro actuator connected to an optical fibre to activate the device. The device will be inserted into the patient’s body in an unexpanded state. One the device reaches the affected site, the probe is expanded by the activation of laser light, and the procedure is completed. This principle founded the basis for the development of a push button blood clot removal device recently introduced to the consumer market.

Recently, research has focused on the development of SMPs that undergo shape change on being subjected to stimuli such as microwaves, ultrasound, electric, and magnetic fields. These SMPs are widely explored for biomedical applications since the instruments capable of providing the stimuli, e.g., ultrasound or UV light source, are already available on the market. In a recent study, ultrasound activated shape memory polymers have been probed for drug delivery applications [101]. A drug delivery container is manufactured using a biodegradable SMP. Once inserted into the body, focused ultrasound is used to non-invasively trigger the container when it reaches the target site. Once triggered, the container undergoes shape change and releases the drugs at the intended site. The discovery will aid target healing in many cases and reduce the requirement for an invasive procedure. Since this study is currently in its elementary phases, not much can be said regarding its long-term effectiveness.

A research group has also probed external radio frequency magnetic field for the wireless actuation of SMP for drug delivery applications [102]. The SMP is attached to a frequency-sensitive wireless heater fabricated using a Cu-clad polyamide. To actuate the SMP, the field frequency is matched with the resonant frequency. The study successfully demonstrated delivery of drug to the targeted site using an RF power of 5 watts. Even though the preliminary results look promising, concerns regarding the biocompatibility of the device need to be addressed.

It is also possible to use SMPs for intragastric implants [94]. When a meal is ingested, the gastric pH changes and returns to the baseline when gastric acid is secreted. The intragastric implants are programmed to expand in response to a change in stomach pH. Once inflated, it provides the patient the satiety of a filled stomach, even after consuming less food. This application of SMPs will help reduce the problem of obesity due to over eating. Other works have also explored using nanocomposites, fluorescent dyes, and conductive materials to develop composites which can also be referred to as shape memory composites (SMCs) [91]. The fillers range from the addition of high modulus organic or inorganic fillers for enhancing the mechanical strength, fluorescent dyes to obtain luminance, magnetic particles to derive magnetic behavior, etc.

A study has compared scaffolds prepared electrospinning of shape memory polyurethane (SMPU) and SMPU with hydroxyapatite nanocomposite additive [103]. The study included a comparison of the scaffolds fabricated from the two materials based on the mechanical strength, porosity and shape memory property. The study concluded that the scaffolds with the additive showed larger diameter, acceptable porosity, higher mechanical strength, and superior shape fixity.

Despite their capabilities and promising results in laboratory studies, SMPs are yet to be explored for real world applications. Once used for commercial applications, more data will be available on the commercial viability and long-term stability of SMP. The factors affecting the usability include the slower response time of shape memory polymers when compared with shape memory alloys [104]. Another factor affecting the usability is the requirement of sterilization in medical devices. US FDA approved sterilization procedures include exposure to radiation, ethylene oxide, or steam. Since these methods potentially impact the function of the SMP, their usage will be limited until a workaround is discovered.

There are many avenues for future advancements in SMPs. The avenues include improving the accuracy of damage recovery in SMPs, reducing the permanent deformation after several cycles, and the development of hybrid SMP capable of being simulated by multiple stimuli, such as light, moisture, as well as electric and magnetic fields [91].

## 6. 3D Printed Polymeric Biomaterials

Additive manufacturing or 3D printing is a manufacturing method adopted for the automated layer-by-layer fabrication of complex geometries using data from a computer-generated model. With 3D printing, it is possible to fabricate biomedical devices and implants that are designed from anatomical data for an individual patient. In the medical field, 3D printing was first used for fabricating anatomical models for dental and medical students, and for pre-surgery planning. However, it is now being explored for the production of craniofacial implants, tissue models for drug delivery, scaffolds for tissue regeneration, and organ printing [105]. The main advantage of 3D printed organs is the reduction of risks associated with organ transplants such as organ rejection [106].

There are numerous types of additive manufacturing technologies, such as material extrusion based fused deposition modelling (FDM), electrospinning, direct ink writing (DIW), photopolymerization based stereolithography (SLA), and powder bed fusion based selective laser sintering (SLS). As a general requirement, the material used for 3D printing should be viscous, structurally stable, biocompatible, should have tunable mechanical properties, form a non-toxic degradation product, and be able to release molecules or drugs. Each 3D printing process also has a specific requirement in terms of properties of materials to be used. In the following sections, we elaborate on the different types of polymeric biomaterials used and some noteworthy works.

### 6.1. Thermoplastics

Thermoplastics are the largely used materials for the 3D printing. Thermoplastics are used in applications that require substantial mechanical strength, long term dimensional stability, chemical inertness, and biocompatibility. Major requirement from thermoplastics used for FDM printing is thixotropic behaviour. Thixotropic thermoplastic filaments are easier to print and provide better accuracy and surface finish. Thermoplastics, such as polycaprolactone (PCL), polyvinyl alcohol (PVA), and polylactic acid (PLA), have been printed either as support material or for direct in vivo implantation [105,107,108].

Biocompatible thermoplastic materials, such as ABS and PLA, are explored and studied for the 3D printing of scaffolds [109]. The scaffolds were studied and compared for their cell ingrowth, viability, and tissue generation. The results showed that both ABS and PLA scaffolds promote cell growth and produce ample matrix within the scaffolds. The scaffolds also provide adequate mechanical stability that is required for tissue engineering. The same research group later probed 3D printed scaffolds printed using POROLAY filament for local doxorubicin delivery in bone metastases secondary to prostate cancer [110]. POROLAY is a proprietary filament that is produced by blending polyurethane and polyvinyl alcohol. Common chemotherapeutics used for cancer treatment when administered globally will have harmful side effects on the body. The scaffolds are used for prolonged low dose treatment and deliver the drugs locally in the tumor affected region.

One of the major factors contributing to the increased penetration of 3D printing in the industry is the availability of low-cost desktop 3D printers. These printers offer the advantages of low investment and are easy to use. One research group explored the possibility of using a desktop 3D printer for the 3D printing of PLA scaffolds [111]. The complete printing process is smooth and requires minimal training. The tests conducted included mechanical testing, test for cytotoxicity, and experiments with osteosarcoma cells. The results of the study place PLA and the 3D printing process favorably for future applications. However, further study will be warranted to optimize the design of the scaffolds. Many works have probed using 3D printed thermoplastics for the fabrication of implants and medical devices. Even though preliminary results of the study look promising, commercial usage will require the fulfilment of stringent requirements concerning medical devices.

### 6.2. 3D Printed Hydrogels

Hydrogels are insoluble polymeric networks with hydrophilic capabilities [112]. Hydrogels swell up to 400% when they come in contact with water. Owing to their excellent biocompatibility and modifiability, they have been widely probed for tissue engineering applications [113]. However, due to their poor mechanical strength and difficulty in printing, most of the hydrogels used for printing are hydrogel composites.

**Figure 14:**
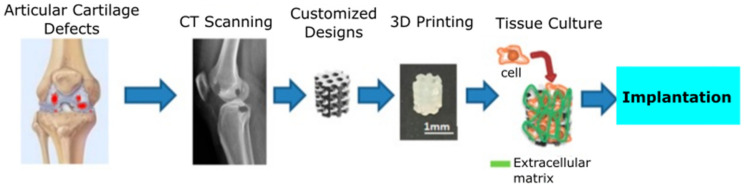
Schematic representation shows the 3D Printing of Water-Based Light-Cured Polyurethane with Hyaluronic Acid scaffolds for Cartilage Tissue Engineering Applications (Reproduced with permission from [114] CC-BY license, 2017).

With articular cartilage diseases affecting millions of people worldwide, one study probed 3D printed cytocompatible hydrogel for tissue engineering applications (Figure 14) [114]. Water-based, light-cured polyurethane with hyaluronic acid was used in 3D print scaffolds that closely mimic the mechanical properties of articular cartilages. A computed tomography (CT) scan is performed for the affected cartilage and customized design is made for the scaffold. The scaffolds are then 3D printed using digital light processing (DLP) technology. The experimental analysis of the scaffolds showed that they facilitate cell adhesion, proliferation, and chondrogenic differentiation.

With a focus on better printability and superior cytocompatibility, highly concentrated Alginate-gellan gum was used for printing scaffolds for tissue engineering [115]. In this study, a mixture of 16.7 wt % alginate and 2 or 3 wt % gellan gum was used for preparation of a hydrogel composite. The printing of scaffolds is undertaken using 3D plotting process. The study included exhaustive analysis of mechanical properties, cytocompatibility and efficiency for tissue engineering applications. The composite showed better printability, superior mechanical properties and improved cell attachment. One drawback of the structure was reduced cell numbers in cell culture over a longer period. However, it is proposed to use divalent cations (Sr^+^ and Zn^+^) to improve cell behavior in long term cell culture.

Even though majority research in the field relies on modifying and optimizing the printing material, research is also being conducted to develop new methods for the 3D printing of hydrogels. A research group has developed a method for 3D printing of hydrogels by freeform reversible embedding of suspended hydrogels (FRESH) [116]. This technology is used to print structures, using hydrogels such as polysaccharide and soft proteins, that are difficult to print using conventional methods. The structures are printed in a suspension of secondary hydrogel that acts as a temporary, thermo reversible and biocompatible support giving them mechanical strength for printing [116]. The printing technology looks promising, particularly due to its high resolution of ~200 µm and low cost [116].

Traditionally, hydrogel composites are prepared by adding composite particles before printing [112]. However, this would sometimes restrict the printability of the hydrogels and also have inconsistent distribution of composite filler particles [117]. In order to ensure a uniform distribution of nanocomposite particles, researchers have developed a novel printing method for freeform 3D printing of hydrogels [117] Using this ‘printing in liquid’ method, researchers have fabricated nanocomposite hydrogel scaffolds using two-step cross-linking of hyaluronic acid-alginate hydrogel inks [117]. An important advantage of the method is that it allows for the addition of ions during the printing process that enhance the mechanical strength and biostability of the printed hydrogels [117].

### 6.3. Bio-Inks

Bioink is used in 3D printing for the preparation of different shaped and sized biomaterials or implants. It is a composite of one or more polymers and it can be in the hydrogel form [118]. Cell aggregates may also be added to the bioink solution for the hybrid combination. Good mechanical, rheological, and biological properties are necessary for the ideal bioink to be used in the 3D printing of biomaterials or organs. Moreover, 3D bioprinting of the aortic valves using alginate/gelatin hydrogel was investigated for the treatment of heart valve disease [119]. Prosthetic devices are commonly used for the treatment of aortic valves and they are found to be imperfect for growing children and adults. In this research, the authors developed 3D printed alginate/hydrogel valve conduits with similar anatomical architecture using a two syringe bioprinter. The hydrogels were encapsulated with dual cell types, namely aortic root sinus smooth muscle cells (SMC) and aortic valve leaflet interstitial cells (VIC). The encapsulated cells were found to be viable within the hydrogel system, even after seven days of culturing. The results of mechanical and in-vitro characterizations demonstrate that the prepared hydrogel valve conduits can be used potentially for the treatment of heart valve disease.

The use of stem cells in the treatment of many incurable diseases has been investigated by many research groups due to their multipotency. A study investigated the use of multipotent articular cartilage-resident chondroprogenitor cells (ACPCs) loaded Gelatin methacryloyl (gelMA) hydrogels for the regeneration and biofabrication of the cartilage tissue [120]. Hydrogels loaded with different cell types, such as ACPCs, bone marrow mesenchymal stromal cells (MSCs), and chondrocytes, were used as a bioink for 3D printing. Upon conducting an exhaustive comparison, ACPCs were found to be better when compared to the other two cell types. The expression of joint lubrication factor PRG4 was also found to be higher in the ACPCs loaded. This study shows the impotence of the ACPCs in articular cartilage regeneration, and it also demonstrates the great potential of future research.

It is important to keep the cell structures intact during the whole 3D printing process. Earlier studies have shown the effect of coating on protecting the cells from the damage that occurs due to the stresses generated during the printing process [121,122]. In a study, adipose derived mesenchymal stem cells were partially coated with silica, inspired by biosilicification [122]. Individual cells were coated with a hard silica layer (called backpack) to provide them with a supporting platform [122]. The two-step process involving bioinspired priming and silicification used Tetra ethyl ortho silicate as the source of silica [122]. Characterization of cells yielded good results in terms of factors such as cell viability and cytocompatibility. This has also generated significant interest for their use in tissue engineering, targeted drug delivery, and biosensing applications [122].

With a similar objective, another study developed a plant-seed inspired cell protection to ensure longer shelf life and better transportability of the cells [121]. The protection, developed using a pyrogallol (PG)-alginate encapsulation system, utilized a pyrogallol- triggered sporulation and germination to protect the mammalian cell [121]. The results of the study in terms of cell viability and improved transportability, coupled with the reproducibility and scalability of the process, make it an interesting avenue for future research [121].

Many studies have worked on the 3D printing of organs, including the cornea [123], retina [124], and kidneys [125]. Despite the promising results of in vitro studies, further testing is warranted for addressing concerns regarding long term viability of the printed organs. Another factor affecting large scale applications of 3D printing in medical applications is the lack of FDA approved materials. Currently, a limited number of 3D printing materials have been approved for use in medical implants which restricts large-scale usage. With the rapid progress in the field and significant interest from all quarters, the next decade may witness significant advancements in this direction.

## 7. Nano-Formulation of Polymeric Biomaterials

Apart from the discussed materials, such as polymeric films, sheets, 3D sponges, and hydrogels, there are other classes of materials, namely 1D and 0D materials. If a material is confined in two directions, such as wires and tubes with a diameter less than 100 nm, it is usually defined as a 1D nanomaterial [126]. Likewise, if the material is confined in all the three dimensions and shows almost spherical shape line dots, micelles with a diameter smaller than 100 nm, then it is called a 0D nanomaterial [127]. Electrospinning of natural and synthetic polymeric materials into nanofibers falls into the class of 1D nanomaterials. Yet, it is very challenging to produce oriented and aligned nanofibers for biomedical applications, such as cellular orientation and attachment (180). In order to achieve an aligned structure of electrospun nanofibers, new developments were established to combine various properties, e.g., size, composition, morphology, and structure [128]. In the biomedical field, these nanofibers can be used to load additional 0D constituents, such as nanoparticles, drugs, and biomolecules, to improve its functionalities. Guided bone regeneration and natural periosteum was achieved through the fabrication of a trilayered membrane of PCL/PU and nano-HA incorporated PU fibers [129]. Synthetic and natural polymer-based nanoparticles have been studied extensively in the field of drug delivery due to their high biocompatibility and biodegradability [130,131]. Many research attempts have been made to achieve the effective therapeutic delivery of the prepared polymeric nanoparticles. Stimuli responsive polymer nanoparticles have also been developed in recent years to improve target-specific therapeutic delivery [132,133]. The nanostructured hierarchical arrangement mimics the structure of extracellular matrix (ECM) and therefore the use of electrospun nanofiber mats is attracting more interest in the field of tissue engineering. Natural (collagen, gelatin and chitosan) and synthetic polymers (PVA and PCL) are studied for their efficiency to prepare the nanofibers [134,135,136,137,138]. The prepared nanofibrous biomaterials have been used mostly in wound healing and drug delivery applications. Table 1 summarizes the properties and applications of different polymeric biomaterials discussed in this manuscript.

## 8. Conclusions and Future Directions

In this review, we briefly described the recent developments made concerning polymer-based biomaterials, with their promising application in the field of tissue engineering and regeneration. Recently developed biomaterials have targeted the controlled and regulated release of active molecules, including in tissue regeneration. The multilayered hydrogels or scaffolds which can release active molecules based on the healing phases or host response are also receiving more attention in the field of tissue engineering. A deeper understanding about the disease/cause is mandatory to develop successful polymer-based biomaterials with desired structure and activity. Polymer-based, stimuli-responsive smart biomaterials represent a highly developing field considering the next generation of biomaterials. Although many attempts have been made for the development of successful biomaterials, a number of challenges remain. It is very difficult to select a polymer for the development of a successful biomaterial. The interaction of host immune cells with the biomaterials presents an unfavorable effect on the final results. The development of patient-specific, 3D-printed, and immunomodulating polymeric biomaterials represents the future of polymer-based biomaterials.

## Figures and Tables

**Table 1:** Category, property and application of the different polymer-based biomaterials.

Material Category	Materials	Property	Application	Reference
Polymeric Films	Poly(acrylic acid) grafted bacterial cellulose	Self-healable film	Wound dressing material	[43]
	Chitosan and konjac glucomannan	Bilayered film	Drug delivery and wound healing	[44]
	Alginate	Bilayered film	Drug delivery and wound healing	[45]
	Sodium alginate, gelatin and reduced graphene oxide	Electrically conductive film	Wound dressing material	[46]
Polymeric Sponges	Agarose and chitosan	3D Scaffold	Liver tissue model	[55]
	Gelatin	Scaffold	Cartilage extracellular matrix	[56]
	Fibroin/chitin/silver nanoparticles	Scaffold	Antibacterial activity	[62]
	Collagen and ZnO nanoparticles	Scaffold	Wound dressing material	[63]
	Gelatin and PVA	Scaffold	Cytocompatible biomaterial for skin regeneration	[64]
	Collagen and hydroxyapatite	Scaffold	Bone tissue engineering	[65]
	Chitosan	Scaffold	Bone tissue engineering	[66]
Hydrogels	Chitosan films cross-linked with epoxy polymers	Shape memory	vascular stents	[99]
	Alginate-gellan gum	3D printing	3D printed scaffolds	[115]
	Collagen	Tissue engineering	Corneal and cardiac tissue engineering	[74]
	poly(ε-caprolactone)-poly(ethylene glycol) and gelatin methacyrylate	Tissue engineering	Corneal tissue engineering	[75]
	Collagen/polyacrylamide	Wound dressing	Wound dressing material	[76]
	Collagen/hyaluronic acid	Tissue engineering	Bone tissue engineering	[77]
	Sodium alginate, Bioglass and poly(lactic-co-glycolic acid)	Multilayered hydrogel	Wound dressing material	[78]
Injectable Hydrogels	Carbon nanotube (CNT) and glycidyl methacrylate functionalized quaternized chitosan	Shape memory	Haemorrhage homeostasis and wound healing	[98]
	Collagen I and III	Tissue engineering	Skin tissue engineering	[80]
	Gelatin/hyaluronic acid	Tissue engineering	Cardiac tissue engineering	[81]
	Gelatin/gold nanoparticles	Tissue engineering	Bone tissue engineering	[82]
Thermoplastics	PLA, calomer	Shape memory	Heat triggered vascular stents	[96,97]
	MM5520 and MM6520	Shape memory	Light triggered vascular stents	[100]
	Cu-clad polyimide	Shape memory	Wireless actuated SMP for drug delivery	[102]
	Polyurethane	Shape memory	Scaffolds with shape memory	[103]
	ABS and PLA	3D printing	3D printed scaffolds	[109,111]
	POROLAY	3D printing	3D printed scaffoldwith drug delivery	[110]
Bioinks	articular cartilage- resident chondroprogenitor cells	3D printing	3D constructs of multiple cell types	[120]
	cell aggregates, natural or synthetic biomaterials	3D printing	Artificial heart valves	[119]

## Data Availability

Not applicable.
